# Chikungunya virus infection in Cynomolgus macaques following Intradermal and aerosol exposure

**DOI:** 10.1186/s12985-017-0804-7

**Published:** 2017-07-20

**Authors:** Chris M. Cirimotich, Eric M. Vela, Jennifer Garver, Roy E. Barnewall, Brian D. Miller, Gabriel T. Meister, James V. Rogers

**Affiliations:** 1 Battelle, West Jefferson, OH 43162 USA; 2 Vaccine and Gene Therapy Institute, Oregon Health and Science University, Portland, OR 97239 USA; 3 Battelle, Columbus, OH 43201 USA

**Keywords:** Chikungunya virus, Alphavirus, Cynomolgus macaque, Aerosol, Intradermal, Animal model

## Abstract

**Background:**

Chikungunya virus (CHIKV) is transmitted via mosquito bite and potentially by aerosol, causing chikungunya fever and arthritic disease in humans. There are currently no licensed vaccines or antiviral therapeutics to protect against CHIKV infection in humans. Animal models recapitulating human disease, especially for transmission by aerosol, are needed for licensure of such medical countermeasures.

**Methods:**

Cynomolgus macaques (CMs) were challenged by intradermal (ID) inoculation or exposure to an aerosol containing CHIKV Ross strain at different target infectious doses (10^3^–10^7^ plaque forming units (PFU)). The clinical and virologic courses of disease were monitored up to 14 days post-exposure.

**Results:**

ID infection of CMs led to overt clinical disease, detectable viremia, and increased blood markers of liver damage. Animals challenged by aerosol exhibited viremia and increased liver damage biomarkers with minimal observed clinical disease. All animals survived CHIKV challenge.

**Conclusions:**

We have described CHIKV infection in CMs following ID inoculation and, for the first time, infection by aerosol. Based on limited reported cases in the published literature, the aerosol model recapitulates the virologic findings of human infection via this route. The results of this study provide additional evidence for the potential use of CMs as a model for evaluating medical countermeasures against CHIKV.

## Background

Chikungunya virus (CHIKV) is a mosquito-transmitted alphavirus (Family *Togaviridae*) that causes chikungunya fever, characterized by a rapid onset of fever coincident with viremia and arthritis. Unlike other alphaviruses that cause arthritogenic disease, such as Barmah Forest, Mayaro, O’nyong-nyong, Sindbis, and Ross River viruses, CHIKV causes large sporadic outbreaks [1, 2]. Originally isolated in 1953 [3], CHIKV caused outbreaks of human disease localized to Africa until the early 21st century [4]. In 2004, larger outbreaks of disease affecting significant proportions of populations occurred on island nations in the Indian Ocean. Epidemic transmission of CHIKV was subsequently identified in India and spread to the Americas in late 2013. Phylogenetic analyses based on complete genomic sequences delineate four lineages of CHIKV: Asian; East, Central, South African (ECSA); Indian Ocean; and West African. Viruses from the Asian and ECSA lineages are likely responsible for outbreaks in the western hemisphere [4].

The four lineages of CHIKVs form a single serotype and are thought to confer cross-protective, lifelong immunity following infection [5]. This suggests effective medical countermeasures can be developed to protect against CHIKV infection and disease. Potential vaccines and therapeutic antibodies are being investigated [6–9], with some entering preclinical development [8, 10]; however, no FDA approved countermeasures currently exist. As countermeasures advance with additional testing and potential licensure, it is important to thoroughly evaluate animal models and transmission routes used for efficacy testing. Improvement of animal models to generate safety and efficacy data for CHIKV vaccine and therapeutic development was a key item identified during a joint World Health Organization- and National Institute of Allergy and Infectious Diseases CHIKV workshop held in 2015 [11].

In humans, productive CHIKV infection can lead to fever and viremia lasting up to 1 week. Myalgia, polyarthritis, and polyarthralgia are also commonly observed in infected individuals, with debilitating polyarthralgia displaying positive predictive value of CHIKV infection in endemic and epidemic areas. A maculopapular rash, similar to that observed during dengue virus infection, may also develop and last for upwards of 1 week [1, 2]. CHIKV infection of non-human primates (NHPs), including rhesus and cynomolgus macaques (CMs), can mirror infection of humans [12, 13]. Studies in NHPs have provided significant insight into the pathogenesis and disease course [14–16], as well as vaccine and therapeutic research and development [6, 17–21]. In a natural history study of CHIKV in CMs, intravenous (IV) inoculation at doses ranging from 10^1^–10^8^ PFU and intradermal (ID) inoculation at a single dose (10^3^ PFU) were evaluated. Clinical observations were dose-dependent with meningoencephalitis and death observed at high doses (10^8^ PFU), fever and rash observed at median doses (10^2^–10^6^ PFU), and no clinical signs observed at the lowest dose evaluated (10^1^ PFU). In contrast, virological findings were consistent across the dose range, with peak viremias of 10^8^–10^9^ viral RNA copies/mL of blood detected 1–3 days post-challenge. The route of inoculation (IV or ID) did not significantly impact either clinical or virological findings [15].

CHIKV is considered a potential biological weapon and bioterrorism agent [1, 22] and laboratory-acquired CHIKV infection by aerosol has been suggested [22–25]; however, this transmission route has not been reported in NHP models and the disease outcome is currently unknown. Here, we aimed to characterize CHIKV infection in CMs infected with a range of challenge doses by ID (closely resembling natural mosquito inoculation) or aerosol challenge. Moreover, this is the first publication to our knowledge characterizing aerosol infection of NHPs with CHIKV.

## Methods

### Animals and virus

Twelve (11 male, 1 female; age range 3–7.5 years old) cynomolgus macaques (CMs; *Macaca fascicularis*) procured from Covance, Inc. (Alice, Texas) were used for this study. Experiments involving animals were approved by the Battelle Institutional Animal Care and Use Committee and were conducted according to procedures outlined in the Guide for the Care and Use of Laboratory Animals, National Research Council.

The ECSA lineage CHIKV strain Ross (ATCC® VR-64™, American Type Culture Collection, Manassas, Virginia) was propagated in Vero E6 cells for use in this study [26]. All experiments involving infectious virus, including cell culture propagation and animal challenges, were performed in the biosafety level-3 facility at the Battelle Biomedical Research Center.

### Study design and animal challenge

CMs were randomized into four groups of two animals and one group of four animals, with each group representing a distinct infection route-dose combination. The groups consisted of ID challenge at one of three target infectious doses (10^3^, 10^5^, or 10^7^ PFU; Groups 1, 2, and 3, respectively) and aerosol challenge at 10^4^ PFU (Group 4; 2 animals) or 10^6^ PFU (Group 5; 4 animals) (Table 1).

**Table 1:** Study design

Group	Number of animals	Infection Route	Infectious Dose
1	2	Intradermal	10^3^
2	2	Intradermal	10^5^
3	2	Intradermal	10^7^
4	2	Aerosol	10^4^
5	4	Aerosol	10^6^

For ID infection, animals were first anesthetized with tiletamine HCl and zolazepam HCl (Telazol; 1–6 mg/kg, intramuscular injection), then injected in the back between the shoulder blades with 0.1 mL of viral inoculum diluted to the target dose.

Prior to animal challenge, aerosolized CHIKV viability and sampling procedures in the large animal exposure system were evaluated by spray factor testing according to the methods of Barnewall and colleagues [27]. Direct challenge of animals via inhalation of CHIKV-containing aerosols was also performed as previously described [27]. Briefly, CMs were anesthetized with Telazol (2–6 mg/kg), placed in a plethysmograph (Buxco Biosystems XA; Buxco Research Systems, Wilmington, NC) within a class III biological safety cabinet, and challenged via head-only aerosol exposure. Aerosol droplets having a mass median aerodynamic diameter that ranged from 1.16–1.29 μm were generated using a three-jet Collison nebulizer (BGI, Waltham, MA). Infectious CHIKV concentrations in aerosol samples collected on gelatin filters were quantified by plaque assay on Vero E6 cells to calculate inhaled doses.

### Animal observations, body weights, and body temperatures

Prior to and following CHIKV challenge, CMs were observed twice daily up to 14 days post-challenge for general health and signs of clinical disease. Baseline body weights were measured 11 days prior to challenge, on the day of challenge, and on every second day after challenge. Animal temperatures were recorded using temperature transponder chips (BioMedic IPTT-300; Bio Medic Data Systems, Inc., Seaford, DE) implanted into the subcutaneous tissue of the shoulder and leg. Baseline temperatures were measured daily for 5 days prior to challenge, approximately 3 h after challenge, and twice daily through day 13 post-challenge.

### Clinical hematology and chemistry

Whole blood samples were collected prior to challenge (baseline) and on days 2, 4, 6, and 8 post-challenge for clinical hematology, and processed to serum for clinical chemistry analyses. Complete blood cell counts and hematology values were measured using an Advia® 120 Hematology Analyzer (Siemens, Deerfield, IL). Clinical chemistry analyses were performed using an Advia® 1200 Chemistry System (Siemens).

### Necropsy

Necropsies were performed on all animals at 14 days post-challenge. Animals were examined for gross lesions and portions of spleen, mesenteric lymph node, liver, and skeletal muscle (~1 cm^3^) were collected for infectious CHIKV quantitation by plaque assay in Vero E6 cells.

### CHIKV plaque assays

Infectious CHIKV titers were determined by plaque assay in Vero E6 cells as follows. Serum samples were diluted directly in cell culture medium without fetal bovine serum (FBS) and tissue samples were homogenized in PBS prior to dilution in cell culture medium without FBS. Serially diluted samples were inoculated in triplicate onto confluent monolayers of Vero E6 cells in 12-well plates and incubated at 37 °C for 1 h with rocking. An overlay containing 1.89% methyl cellulose in cell culture medium was then added. At 72 h post-challenge, the overlay was removed and cells were stained using a 1.3 g/L crystal violet solution in formalin. The average virus titer was calculated for each sample containing 3–150 plaques in each well. The lower limit of quantification was 2.48 log_10_ PFU/mL (300 PFU/mL).

### Statistical analysis

Randomization of animals into groups and all statistical analyses were performed using SAS® (Version 9.3; SAS Institute, Cary, NC). Significant shifts from baseline at each study day were determined for each group by the following ANOVA model fitted separately for each parameter at specified study days:

Y_*dij*_ – Y_*bij*_ = μ + Group_*i*_ + ε_*ij*_ *.*

where Y_*dij*_ is the observed parameter result for the *j*th animal in Group *i* (*i* = vehicle, low, middle-low, middle-high, high) on Study Day *d*, Y_*bij*_ is the observed parameter result for the *j*th animal in Group *i* at baseline, μ is an overall constant, Group_*i*_ is the effect of Group *i*, and ε_*ij*_ is the random error left unexplained by the model. Least square mean estimates from these models were calculated and approximate t-tests were performed to determine if, for each group, there was a significant shift from baseline at each study day. Tukey’s multiple comparisons were performed to determine which pairs of groups were significantly different. All results are reported at the 0.05 level of significance.

## Results

### Animal challenge

Twelve CMs were challenged with CHIKV by either ID inoculation or by aerosol. For ID challenge, animals were inoculated with 10^3^, 10^5^, or 10^7^ PFU (Groups 1, 2, and 3, respectively; Table 1), with two animals per dose group. For challenge by aerosol, spray factor testing performed prior to challenge demonstrated that aerosol sampling with gelatin filters successfully recovered infectious CHIKV (data not shown). Therefore, this sampling method was used for analysis of inhaled doses. CMs were challenged by head-only exposure with one of two target doses of aerosolized CHIKV. Inhaled doses for the 10^4^ and 10^6^ PFU groups (Groups 4 and 5, respectively; Table 1) averaged 2.92 × 10^4^ and 3.33 × 10^6^ PFU per animal, respectively.

### Clinical disease

All CMs challenged with CHIKV were observed twice daily for signs of virus infection. Clinical signs of overt chikungunya fever, including hunched posture, ataxia, and shivering, were observed in 100% of ID-challenged animals, regardless of infectious dose. Signs were first observed in these animals at 1–3 days post-challenge and persisted through day 8 post-challenge. In contrast, clinical disease was observed in only one of six (17%) CMs challenged by aerosol, an animal in Group 4. This animal showed overall signs of discomfort 2 days post-challenge and hunched posture with shivering from three to 5 days post-challenge. No animals in the high-dose aerosol group (Group 5) displayed outward signs of clinical disease. Signs of neurologic disease, reported for high-dose (10^8^ PFU) CHIKV infection in CMs by IV injection [15], were not observed in this study. All challenged animals survived to the end of study.

### Body weights and temperatures

**Fig. 1:**
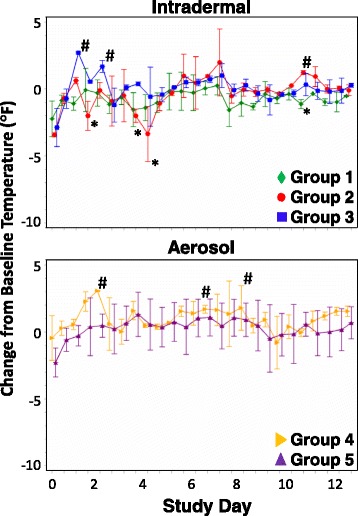
Change from baseline body temperature following challenge of CMs with CHIKV. Body temperatures of CMs challenged by ID inoculation (top graph) or by aerosol (bottom graph) were recorded twice daily for 13 days. Mean changes from baseline temperature are shown with standard normal 95% confidence intervals for each group. * - group mean temperature significantly decreased compared to baseline count at 0.05 level; # - group mean temperature significantly increased compared to baseline count at 0.05 level

The body weights of all animals, measured every 5 days post-challenge, remained constant compared to pre-challenge weight (data not shown). Animal body temperature changes were variable throughout the study period (Fig. 1); however, minimal group effect was observed. Animals in Group 3 showed significant mean temperature increases on days 1 and 2 post-challenge; the increased temperatures on day 1 were significantly greater than all other study groups (Tukey’s *p*-values 0.0013, 0.0364, 0.0308, and 0.0024 when compared to Groups 1, 2, 4, and 5, respectively). Animals in Group 4 had significant mean temperature increases on day 2 post-challenge that were higher than all groups except Group 3 (Tukey’s *p*-values 0.0092, 0.0128, and 0.0171 when compared to Groups 1, 2, and 5, respectively). Group 2 had significant mean temperature decreases on day 4 post-challenge. With few exceptions, temperatures remained near baseline for the remainder of the study in all groups after day 4 post-challenge (Fig. 1).

### Viremia and virus titers in tissues

Serum samples collected every 2 days post-challenge were assessed for infectious CHIKV loads by plaque assay (Table 2). Qualitatively, all 12 animals displayed detectable viremia at one or more timepoints following challenge. Peak viremia for 10/12 (83%) animals occurred on day two post-challenge, with six of these animals showing sustained viremia between 2 and 4 days post-challenge. A single animal in Group 5 had detectable viremia only at day 4 post-challenge. Five of six (83%) aerosol-challenged animals had detectable viremia on both days as compared to only two of six (33%) animals challenged by ID inoculation. No animals in Groups 2 or 3 had detectable viremia on day 4 post-challenge (Table 2).

**Table 2:** Summary of CHIKV viremia results

Group	Challenge	Infectious Dose	Day 2 (Log_10_ PFU/mL)		Day 4 (Log_10_ PFU/mL)		Day 6 (Log_10_ PFU/mL)	
			Titer	Average	Titer	Average	Titer	Average
1	Intradermal	10^3^	5.93	6.37	3.06	2.96	<2.48^a^	<2.48^a^
			6.80		2.86		<2.48^a^	
2	Intradermal	10^5^	4.75	5.27	<2.48^a^	<2.48^a^	<2.48^a^	<2.48^a^
			5.78		<2.48^a^		<2.48^a^	
3	Intradermal	10^7^	5.15	5.30	<2.48^a^	<2.48^a^	<2.48^a^	<2.48^a^
			5.44		<2.48^a^		<2.48^a^	
4	Aerosol	10^4^	5.23	5.25	3.60	3.80^b^	<2.48^a^	<2.48^a^
			5.26		4.00		<2.48^a^	
5	Aerosol	10^6^	3.81	3.38^c^	3.12	2.81	<2.48^a^	<2.48^a^
			2.67		2.88		<2.48^a^	
			<2.48^a^		2.60		<2.48^a^	
			3.67		2.65		<2.48^a^	

^a^Plaque assay Lower Limit of Quantification = 2.48 log_10_ PFU/mL

^b^Significantly higher than all other groups at this timepoint at the 0.05 level

^c^Significantly lower than all other groups at this timepoint at the 0.05 level

Quantitatively, the highest viremia titers at days 2 and 4 post-challenge were observed in animals challenged by either route with the lowest target doses of CHIKV (Groups 1 and 4). The highest titers on days 2 and 4 post-challenge were observed in Group 1 (average 6.37 log_10_ PFU/mL) and Group 4 (average 3.80 log_10_ PFU/mL), respectively. Day 2 mean viremia titers in Groups 1, 2, 3, and 4 were significantly higher than Group 5 titers (Tukey’s *p*-values 0.0035, 0.0313, 0.0293, and 0.0329, respectively), while Group 4 mean titers were significantly higher than all other groups on day 4 (Tukey’s *p*-values 0.0226, 0.0005, 0.0005, and 0.0042 compared to Groups 1, 2, 3, and 5, respectively). No animals had detectable viremia after day 4 post-challenge (Table 2).

Sections of liver, spleen, skeletal muscle, and mesenteric lymph node collected at necropsy (14 days post-challenge) from all animals were also evaluated for CHIKV. No infectious virus (PFU) was detected in these tissues in any of the challenged animals (data not shown).

### Clinical pathology

**Fig. 2:**
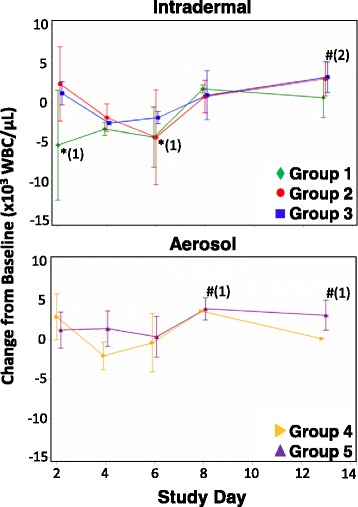
Change from baseline WBC counts following challenge of CMs with CHIKV. WBC counts of CMs challenged by ID inoculation (top graph) or by aerosol (bottom graph) were determined on days 2, 4, 6, 8, and 13 post-challenge. Mean changes from baseline WBC counts are shown with standard normal 95% confidence intervals for each group. * - group mean WBC count significantly decreased compared to baseline at 0.05 level; # - group mean WBC count significantly increased compared to baseline at 0.05 level; numbers in parentheses represent the number of groups significantly different from baseline at that time point

**Fig. 3:**
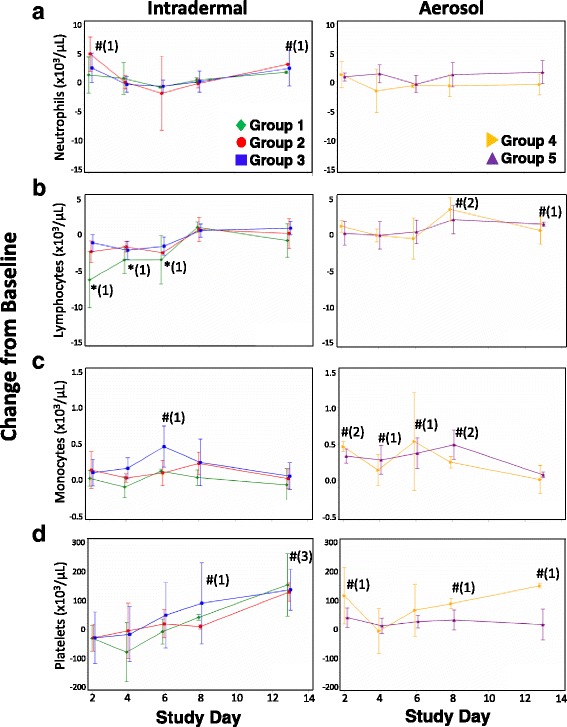
Change from baseline in various blood cell counts following challenge of CMs with CHIKV. Counts of (**a**) neutrophils, (**b**) lymphocytes, (**c**) monocytes, and (**d**) platelets in CMs challenged by ID inoculation (left graphs) or by aerosol (right graphs) were determined on days 2, 4, 6, 8, and 13 post-challenge. Mean changes from baseline blood cell counts are shown with standard normal 95% confidence intervals for each group. * - group mean parameter significantly decreased compared to baseline at 0.05 level; # - group mean parameter significantly increased compared to baseline at 0.05 level; numbers in parentheses represent the number of groups significantly different from baseline at that time point

**Fig. 4:**
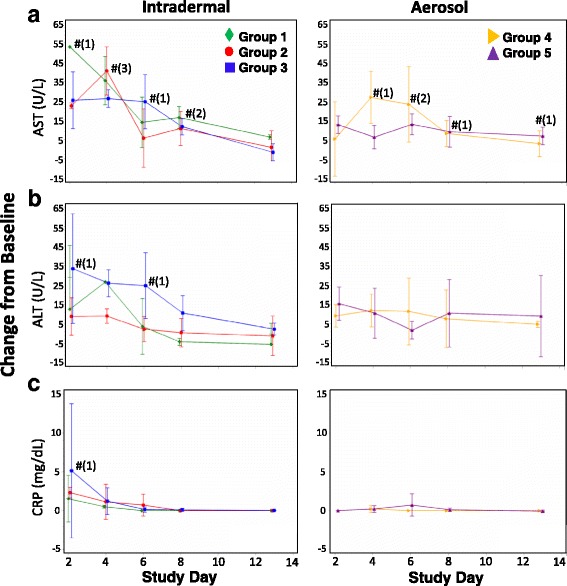
Change from baseline in clinical chemistry parameters following challenge of CMs with CHIKV. Concentrations of **a** AST, **b** ALT, and **c** CRP in CMs challenged by ID inoculation (left graphs) or by aerosol (right graphs) were determined on days 2, 4, 6, 8, and 13 post-challenge. Mean changes from baseline parameter concentrations are shown with standard normal 95% confidence intervals for each group. * - group mean parameter significantly decreased compared to baseline at 0.05 level; # - group mean parameter significantly increased compared to baseline at 0.05 level; numbers in parentheses represent the number of groups significantly different from baseline at that time point

Blood cell and blood chemistry parameters were analyzed in samples collected on Days 2, 4, 6, 8, and 13 post-challenge and compared to baseline. White blood cell (WBC) counts (Fig. 2), additional blood cell counts (Fig. 3), and multiple clinical chemistry parameters (Fig. 4) were analyzed.

WBC counts were variable during the study period. Mean shifts from WBC baseline counts were significantly lower in Group 1 on days 2 and 4 post-challenge. Counts were near baseline for all other groups on day 2, and decreased compared to baseline on days 4 and 6 post-challenge in Groups 1, 2, 3, and 4. By day 13 post-challenge, mean WBC counts were near or elevated above baseline in all groups, with Groups 2, 3, and 5 displaying significantly elevated counts (Fig. 2).

In ID-challenged groups (Groups 1 to 3) at day 2 post-challenge, neutrophil counts were above baseline, with Group 2 showing significantly increased counts (Fig. 3a). Neutrophil counts then decreased to baseline levels by day 4 post-challenge and remained near baseline until day 13. In aerosol-challenged groups, neutrophil counts remained near baseline for the entire study period (Fig. 3a). From day 2 to day 6 post-challenge, Groups 1, 2, and 3 showed decreased lymphocyte counts, with Group 1 showing significantly decreased counts (Fig. 3b). Lymphocyte counts in these animals remained below baseline until day 8 post-challenge. In contrast, aerosol-challenged animals (Groups 4 and 5) showed near-baseline lymphocyte counts throughout the study (Fig. 3b). Monocyte counts in ID-challenged animals remained near baseline except for a significant increase above baseline for Group 3 on day 6 post-challenge (Fig. 3C). Group 4 monocyte counts were significantly above baseline on days 2, 6, and 8 post-challenge, and counts for Group 5 were significantly above baseline on days 2, 4, and 8 post-challenge.

Platelet counts were variable. Group 1 animals showed a decrease in platelets on day 4 post-challenge, with counts increasing throughout the remainder of the study. Group 2 and 3 mean platelet counts increased throughout the study. Platelet counts in Group 4 were significantly above baseline at day 2 post-challenge, decreased below baseline at day 4, and then increased through day 13. Group 5 mean platelet counts remained near baseline throughout the entire study period. Except for Group 5, all groups had mean platelet counts significantly above baseline at day 13 (Fig. 3d).

CHIKV infection resulted in changes to clinical chemistry parameters, including aspartate aminotransferase (AST), alanine aminotransferase (ALT), and C-reactive protein (CRP) (Fig. 4). All groups displayed mean AST increases from baseline from days 2 to 8 post-challenge. On day 2 post-challenge, levels were significantly increased above baseline in Group 2. On day 4, levels in Groups 1–4 were significantly elevated. Levels in Groups 3, 4, and 5 were significantly elevated on day 6, and in Groups 1, 3, and 5 on day 8 post-challenge (Fig. 4a). Mean ALT levels were elevated in all groups until at least day 6 post-challenge, with significantly increased levels above baseline in Group 3 on days 2 and 6 post-challenge (Fig. 4).

Levels of CRP, an acute-phase reactant used as an indicator for inflammation, were elevated in some animals in Groups 1, 2, and 3 for at least 4 days following ID challenge. Peak CRP levels in ID-challenged animal groups were reached at day 2, with animals in Group 3 presenting the highest mean levels. In these groups, CRP levels then returned to near-baseline by day 6 and through the remainder of the study. Elevated CRP levels were not observed in groups challenged by aerosol (Fig. 4c).

## Discussion

This study aimed to provide a preliminary characterization of chikungunya disease in CMs challenged by ID inoculation or by aerosol with CHIKV. The ID inoculation route closely mimics natural human infection via mosquito bite, while aerosol exposure has also been proposed as a potential transmission route in humans [22–24]. Infectious doses at several concentrations were evaluated to better characterize these routes for development of CHIKV infection models in CMs.

In natural outbreaks of chikungunya fever, presumably transmitted through the bite and inoculation of virus by infected mosquitoes, an incubation period of 2–6 days results in rapid onset of fever, arthralgia, headache, weakness, and rash during a viremic period that typically lasts for 5–7 days [2]. Leukopenia, thrombocytopenia, and elevated liver and muscle enzymes are also common laboratory findings [28, 29].

Transmission by inhalation of infected aerosols has been hypothesized for laboratory-acquired infections [22–25] and CHIKV is the only arthritogenic alphavirus suggested to be transmitted by this route [1, 24]; CHIKV is considered a potential agent for bioterrorism due to this putative transmission [1, 22]. Although the number of cases is limited, the signs and symptoms following suspected aerosol exposure are mild and of shorter duration than those following mosquito transmission [1]. In one case report, the infected adult male presented with a mild fever for 3 days, myalgia, headache, leukocytosis, and a maculopapular rash following an estimated eight-day incubation period. Serum viremia was detectable at 18 h after suspected time of disease onset, but was not detectable by 72 h post-onset [23]. In another potential human infection by aerosol, viremia was detected in a laboratory worker approximately 24 h after unspecified symptoms were observed, an estimated 6 days after the suspected exposure event (aerosolization of dried brain powder derived from an infected mouse) [25]. In the current study, our observations in CMs following exposure to aerosols containing CHIKV corroborated the data published on human infection via this route, such as mild or no overt clinical disease with productive infection via detectable, sustained viremia [23, 25].

The ID route of CHIKV inoculation in CMs has been reported previously, using the CHIKV isolate LR2006-OPY1 (Indian Ocean lineage [26]) at a single infectious dose (10^3^ PFU) [15]. In the present study, ID challenge with the CHIKV Ross strain promoted similarities and differences to those observed using the CHIKV isolate LR2006-OPY1 [15]. In both studies, peak viremia was reached by day 2 post-challenge, which was coincident with leukopenia, and animals exhibited elevated levels of enzymes indicative of liver or muscle injury (AST, ALT) [15]. These observations are common early clinical findings in humans infected with CHIKV [29]. Peak viremia coincident with leukopenia was also observed following intravenous infection of CMs [15] and subcutaneous infection of rhesus macaques [14]. However, prolonged viremia and fever were not observed in ID-challenged animals in the present study. Differences in sensitivity between the virus detection methods used (infectious virus detection through plaque assay compared to quantitative RT-PCR by Labadie and colleagues [15]) may at least partially explain the variances in viremia observed, as well as the lack of CHIKV detection in tissues in our studies compared to detectable viral genomes in tissues described previously [15]. The CHIKV E1 glycoprotein-targeted RT-PCR assay used by Labadie and colleagues has not been correlated with infectious CHIKV titers [30]; however, viral genome copy number detected with an NSP1-targeted quantitative RT-PCR displayed high correlation with infectious virus titers [31], suggesting the two assays may be equally sensitive. A more likely reason for the observed differences in viral burden and fever is the use of CHIKV strains from distinct lineages (the ECSA lineage Ross strain here compared to the Indian Ocean lineage LR2006-OPY1 strain [15]) for challenge. CHIKV strains from disparate lineages have shown virulence differences in multiple animal models. The LR2006-OPY1 strain was more virulent than an Asian lineage strain in adult mice [32] and an Asian lineage strain was more neurovirulent than an ECSA lineage strain in suckling mice [33]. In rhesus macaques, the West African CHIKV strain 37,997 produced significantly higher viremia than an epidemic ECSA lineage strain [14], but was as virulent as the LR2006-OPY1 strain [16]. Extrapolation of results from these NHP studies suggests the LR2006-OPY1 strain may be more virulent in NHPs than the ECSA lineage Ross strain used in the present study.

Even with the limited number of animals used in our investigations, ID inoculation and challenge by aerosol resulted in noticeably different clinical presentation yet similar virologic findings. All ID-challenged animals were overtly ill based on clinical observations and many showed abnormal clinical hematology (Figs. 3 and 4), while aerosol-challenged CMs showed minimal clinical evidence of disease. For both infection routes, CHIKV viremia peaked by day 2 post-challenge and was undetectable by day 6 post-challenge. Other studies comparing CHIKV infection routes in NHPs have shown that CM infection by IV and ID routes resulted in similar clinical and virologic findings, with detectable CHIKV RNA in the blood until at least day 6 post-challenge [15]. Infection of Indian bonnet macaques (*Macaca radiata*) via mosquito bite generally resulted in delayed detectable and peak viremia compared to IV inoculation [34].

With both challenge routes investigated in this study, higher viremia titers were observed in CMs exposed to lower infectious doses of virus (Table 2). Animals in Group 1, exposed to 10^3^ PFU by ID inoculation, had higher CHIKV titers than Groups 2 and 3 (10^5^ and 10^7^ PFU inoculation doses, respectively), and animals in Group 4, exposed to 10^4^ PFU by aerosol, had higher titers than Group 5 (10^6^ PFU inoculation dose). These trends were consistent on both days 2 and 4 post-challenge. Similar to other studies [15, 16], viremia kinetics were infectious dose-independent in our study.

One possible explanation for the apparent inverse correlation observed between infectious dose and viremia titers is the innate immune response mounted immediately following infection. Production of type I interferons (IFNs), responsible for establishing an overall antiviral state in the infected host through the induction of hundreds of antiviral protein genes, is stimulated in CHIKV-infected humans [35–39] and CMs [15] during the acute phase of infection. A positive correlation was identified for viremia titers and IFN-α levels in human serum samples collected during the CHIKV outbreak in La Reunion during 2005–2006, and increasing CHIKV multiplicity of infection was shown to increase IFN-β secretion in cultured human fibroblasts [35]. Additionally, individuals with higher levels of viremia (mean viral load, 1.31 × 10^8^ PFU/mL) at time of hospital admission had significantly higher levels of IFN-α than individuals with lower viremia levels (mean viral load, 1.95 × 10^4^ PFU/mL) [38]. In CMs infected IV with 10^3^ PFU of CHIKV strain LR-2006 OPY1, a spike in IFN-α/β levels observed 2 days post-challenge was coincident with peak viremia levels; IFN levels returned to baseline by day four post-challenge [15]. These studies suggest induction of type I IFNs by CHIKV infection is virus concentration-dependent. It is possible that the higher challenge doses in our study similarly stimulate a more-robust antiviral response (through IFN induction) than the lower challenge doses, leading to reduced virus replication and subsequent lower viremia titers. A direct correlation was detected between viremia (measured in viral RNA copies/mL) and challenge dose in CMs challenged by IV inoculation of CHIKV; however, the different virus strain, inoculation route, and viremia quantification methods could account for the differences observed between the two studies [15]. Additional experiments directly comparing IFN levels and induction of IFN-stimulated genes at early timepoints following CM challenge with different doses of CHIKV would be required to investigate the potential role of this antiviral response in limiting viremia titers in groups challenged with higher infectious doses. If this hypothesis holds true, vaccination and therapeutic strategies able to maximize type I IFN induction may be efficacious against CHIKV infection.

## Conclusions

We have shown that ID and aerosol challenge of CMs with CHIKV leads to productive infection with presentations that mimic reported symptoms of human infection with the virus by these two routes. Further investigation with larger numbers of animals could refine these models, which will be imperative for evaluation and eventual approval of countermeasures against CHIKV infection in humans.

## Abbreviations

ALT: Alanine aminotransferase
AST: Aspartate aminotransferase
CHIKV: Chikungunya virus
CM: Cynomolgus macaque
CRP: C-reactive protein
ECSA: East, Central, South Africa
LUC: Large unstained cell
NHP: Non-human primate
NSP1: Nonstructural protein 1
PFU: Plaque-forming unit
RNA: Ribonucleic acid
RT-PCR: Reverse transcriptase-polymerase chain reaction
WBC: White blood cell

## References

[1] Suhrbier A, Jaffar-Bandjee MC, Gasque P (2012). Arthritogenic alphaviruses--an overview. Nat Rev Rheumatol.

[2] Weaver SC, Lecuit M (2015). Chikungunya virus and the global spread of a mosquito-borne disease. N Engl J Med.

[3] Ross RW (1956). The Newala epidemic. III. The virus: isolation, pathogenic properties and relationship to the epidemic. J Hyg (Lond).

[4] Weaver SC, Forrester NL (2015). Chikungunya: evolutionary history and recent epidemic spread. Antivir Res.

[5] Weaver SC, Osorio JE, Livengood JA, Chen R, Stinchcomb DT (2012). Chikungunya virus and prospects for a vaccine. Expert Rev Vaccines.

[6] Erasmus JH, Auguste AJ, Kaelber JT, Luo H, Rossi SL, Fenton K, Leal G, Kim DY, Chiu W, Wang T (2016). A chikungunya fever vaccine utilizing an insect-specific virus platform. Nat Med.

[7] Goo L, Dowd KA, Lin TY, Mascola JR, Graham BS, Ledgerwood JE, Pierson TC (2016). A virus-like particle vaccine elicits broad neutralizing antibody responses in humans to all Chikungunya virus genotypes. J Infect Dis.

[8] Schwameis M, Buchtele N, Wadowski PP, Schoergenhofer C, Jilma B (2016). Chikungunya vaccines in development. Hum Vaccin Immunother.

[9] Smith SA, Silva LA, Fox JM, Flyak AI, Kose N, Sapparapu G, Khomandiak S, Ashbrook AW, Kahle KM, Fong RH (2015). Isolation and characterization of broad and Ultrapotent human monoclonal antibodies with therapeutic activity against Chikungunya virus. Cell Host Microbe.

[10] Smalley C, Erasmus JH, Chesson CB, Beasley DW (2016). Status of research and development of vaccines for chikungunya. Vaccine.

[11] Graham BS, Repik PM, Yactayo S (2016). Chikungunya in the Americas: recommendations and conclusions. J Infect Dis.

[12] Broeckel R, Haese N, Messaoudi I, Streblow DN (2015). Nonhuman primate models of Chikungunya virus infection and disease (CHIKV NHP model). Pathogens.

[13] Haese NN, Broeckel RM, Hawman DW, Heise MT, Morrison TE, Streblow DN (2016). Animal models of Chikungunya virus infection and disease. J Infect Dis.

[14] Chen CI, Clark DC, Pesavento P, Lerche NW, Luciw PA, Reisen WK, Brault AC (2010). Comparative pathogenesis of epidemic and enzootic Chikungunya viruses in a pregnant rhesus macaque model. Am J Trop Med Hyg.

[15] Labadie K, Larcher T, Joubert C, Mannioui A, Delache B, Brochard P, Guigand L, Dubreil L, Lebon P, Verrier B (2010). Chikungunya disease in nonhuman primates involves long-term viral persistence in macrophages. J Clin Invest.

[16] Messaoudi I, Vomaske J, Totonchy T, Kreklywich CN, Haberthur K, Springgay L, Brien JD, Diamond MS, Defilippis VR, Streblow DN (2013). Chikungunya virus infection results in higher and persistent viral replication in aged rhesus macaques due to defects in anti-viral immunity. PLoS Negl Trop Dis.

[17] Akahata W, Yang ZY, Andersen H, Sun S, Holdaway HA, Kong WP, Lewis MG, Higgs S, Rossmann MG, Rao S, Nabel GJ (2010). A virus-like particle vaccine for epidemic Chikungunya virus protects nonhuman primates against infection. Nat Med.

[18] Harrison VR, Eckels KH, Bartelloni PJ, Hampton C (1971). Production and evaluation of a formalin-killed Chikungunya vaccine. J Immunol.

[19] Mallilankaraman K, Shedlock DJ, Bao H, Kawalekar OU, Fagone P, Ramanathan AA, Ferraro B, Stabenow J, Vijayachari P, Sundaram SG (2011). A DNA vaccine against chikungunya virus is protective in mice and induces neutralizing antibodies in mice and nonhuman primates. PLoS Negl Trop Dis.

[20] Nakao E, Hotta S (1973). Immunogenicity of purified, inactivated chikungunya virus in monkeys. Bull World Health Organ.

[21] Pal P, Dowd KA, Brien JD, Edeling MA, Gorlatov S, Johnson S, Lee I, Akahata W, Nabel GJ, Richter MK (2013). Development of a highly protective combination monoclonal antibody therapy against Chikungunya virus. PLoS Pathog.

[22] Frolov IV, Weaver SC, Wang E: Chimeric chikungunya virus and uses thereof. vol. US20110171249A1. pp. 22. USA; 2011:22.

[23] Shah KV, Baron S (1965). Laboratory infection with chikungunya virus: a case report. Indian J Med Res.

[24] Tesh RB (1982). Arthritides caused by mosquito-borne viruses. Annu Rev Med.

[25] McIntosh BM, Paterson HE, Donaldson JM, De Sousa J (1963). Chikungunya virus: viral susceptibility and transmission studies with some vertebrates and mosquitoes. S Afr J Med Sci.

[26] Volk SM, Chen R, Tsetsarkin KA, Adams AP, Garcia TI, Sall AA, Nasar F, Schuh AJ, Holmes EC, Higgs S (2010). Genome-scale phylogenetic analyses of chikungunya virus reveal independent emergences of recent epidemics and various evolutionary rates. J Virol.

[27] Barnewall RE, Fisher DA, Robertson AB, Vales PA, Knostman KA, Bigger JE (2012). Inhalational monkeypox virus infection in cynomolgus macaques. Front Cell Infect Microbiol.

[28] Borgherini G, Poubeau P, Jossaume A, Gouix A, Cotte L, Michault A, Arvin-Berod C, Paganin F (2008). Persistent arthralgia associated with chikungunya virus: a study of 88 adult patients on reunion island. Clin Infect Dis.

[29] Simon F, Parola P, Grandadam M, Fourcade S, Oliver M, Brouqui P, Hance P, Kraemer P, Ali Mohamed A, de Lamballerie X (2007). Chikungunya infection: an emerging rheumatism among travelers returned from Indian Ocean islands. Report of 47 cases. Medicine (Baltimore).

[30] Laurent P, Le Roux K, Grivard P, Bertil G, Naze F, Picard M, Staikowsky F, Barau G, Schuffenecker I, Michault A (2007). Development of a sensitive real-time reverse transcriptase PCR assay with an internal control to detect and quantify chikungunya virus. Clin Chem.

[31] Carletti F, Bordi L, Chiappini R, Ippolito G, Sciarrone MR, Capobianchi MR, Di Caro A, Castilletti C (2007). Rapid detection and quantification of Chikungunya virus by a one-step reverse transcription polymerase chain reaction real-time assay. Am J Trop Med Hyg.

[32] Teo TH, Her Z, Tan JJ, Lum FM, Lee WW, Chan YH, Ong RY, Kam YW, Leparc-Goffart I, Gallian P (2015). Caribbean and la Reunion Chikungunya virus isolates differ in their capacity to induce Proinflammatory Th1 and NK cell responses and acute joint pathology. J Virol.

[33] Chiam CW, Chan YF, Ong KC, Wong KT, Sam IC (2015). Neurovirulence comparison of chikungunya virus isolates of the Asian and east/central/south African genotypes from Malaysia. J Gen Virol.

[34] Paul SD, Singh KR (1968). Experimental infection of Macaca Radiata with Chikungunya virus and transmission of virus by mosquitoes. Indian J Med Res.

[35] Schilte C, Couderc T, Chretien F, Sourisseau M, Gangneux N, Guivel-Benhassine F, Kraxner A, Tschopp J, Higgs S, Michault A (2010). Type I IFN controls chikungunya virus via its action on nonhematopoietic cells. J Exp Med.

[36] Wauquier N, Becquart P, Nkoghe D, Padilla C, Ndjoyi-Mbiguino A, Leroy EM (2011). The acute phase of Chikungunya virus infection in humans is associated with strong innate immunity and T CD8 cell activation. J Infect Dis.

[37] Ng LF, Chow A, Sun YJ, Kwek DJ, Lim PL, Dimatatac F, Ng LC, Ooi EE, Choo KH, Her Z (2009). IL-1beta, IL-6, and RANTES as biomarkers of Chikungunya severity. PLoS One.

[38] Chow A, Her Z, Ong EK, Chen JM, Dimatatac F, Kwek DJ, Barkham T, Yang H, Renia L, Leo YS, Ng LF (2011). Persistent arthralgia induced by Chikungunya virus infection is associated with interleukin-6 and granulocyte macrophage colony-stimulating factor. J Infect Dis.

[39] Venugopalan A, Ghorpade RP, Chopra A (2014). Cytokines in acute chikungunya. PLoS One.

